# Stool-based SDC2/SFRP2/TFPI2 methylation assay for colorectal neoplasia screening: a multicenter, case-control study

**DOI:** 10.3389/fonc.2026.1748759

**Published:** 2026-01-30

**Authors:** Xiaoping Li, Qiuyu Liao, Wenhui Yang, Zhiguo Xiong, Mark Holmes, Kaiyu Li, Jie Pan, Jing Yan, Gang Liu

**Affiliations:** 1 Department of General Surgery, Tianjin Medical University General Hospital, Tianjin, China; 2 Ambulatory Surgery Center, Tianjin Medical University General Hospital, Tianjin, China; 3 Tianjin Key Laboratory of Precise Vascular Reconstruction and Organ Function Repair, Tianjin, China; 4 Tianjin General Surgery Institute, Tianjin, China; 5 Department of Gastroenterology, Shanxi Province Cancer Hospital, Shanxi Hospital Affiliated to Cancer Hospital, Chinese Academy of Medical Sciences, Cancer Hospital Affiliated to Shanxi Medical University, Taiyuan, Shanxi, China; 6 Department of Gastrointestinal Surgery, Hubei Cancer Hospital, Tongji Medical College, Huazhong University of Science and Technology, Wuhan, Hubei, China; 7 Department of Veterinary Medicine, University of Cambridge, Cambridge, United Kingdom; 8 Department of Gastroenterology, Wenzhou Central Hospital, The Dingli Clinical College of Wenzhou Medical University, The Second Affiliated Hospital of Shanghai University, Wenzhou, Zhejiang, China; 9 Holosensor Medical Technology Ltd, Suzhou, Jiangsu, China

**Keywords:** colorectal neoplasia, detection, DNA methylation, screening, SDC2/SFRP2/TFPI2, stool

## Abstract

**Introduction:**

Colorectal neoplasia is a significant global health burden. Early detection is critical for improving clinical outcomes. However, developing a noninvasive strategy for routine detection of colorectal neoplasia remains challenging. This study evaluated the diagnostic performance of a combined SDC2/SFRP2/TFPI2 gene methylation stool DNA test for detecting colorectal cancer (CRC) and advanced adenoma (AA).

**Methods:**

A multicenter, case-control study was conducted involving 409 patients with CRC, 82 patients with AA, and 495 control participants between July 2022 and March 2024. Stool samples were collected from all participants and analyzed for SDC2, SFRP2, and TFPI2 methylation using quantitative fluorescent polymerase chain reaction (PCR). To evaluate the detection efficacy of the SDC2/SFRP2/TFPI2 methylation assay in comparison to conventional methods, fecal occult blood test (FOBT) and serum carcinoembryonic antigen (CEA) levels were measured in 110 CRC patients and 100 healthy individuals at Tianjin Medical University General Hospital.

**Results:**

The stool DNA test demonstrated high sensitivity for CRC (94.13%, 95% CI: 91.42-96.03) and AA (59.76%, 95% CI: 48.94-69.70), with 94.74% specificity (95% CI: 90.30-97.21) in the normal controls. Notably, the SDC2 marker displayed zero false positives among 98 digestive system cancer cases within the control groups, while SFRP2 and TFPI2 showed 8.16% and 10.2% false-positive rates, respectively. The stool DNA test accurately identified CRC, with an area under the receiver operating characteristic (ROC) curve (AUC) of 0.9343, and AA, with an AUC of 0.7729. Furthermore, positive stool DNA test results were significantly associated with distal cancer, ulcerative type, deeper tumor infiltration, flat-type AA, and lesions with high-grade dysplasia (P < 0.05). Among the 110 patients with CRC and 100 healthy controls, the AUC values for FOBT, CEA, and stool DNA were 0.7532, 0.6732, and 0.9399, respectively. The integration of all three detection methods achieved the highest detection efficacy (AUC = 0.9725).

**Conclusion:**

The combined methylation assay of SDC2, SFRP2, and TFPI2 outperformed FOBT and CEA in detecting CRC and AA, although further optimization is required for AA detection. Its high specificity and compatibility with routine screening procedures underscore its potential as a tool for detecting colorectal neoplasia.

## 1 Introduction

Colorectal cancer (CRC) is the third most common cancer worldwide, with an estimated 1.93 million new diagnoses and over 900, 000 deaths in 2022 (1). The etiology of CRC remains unclear, and patients typically present with atypical symptoms. Moreover, most cases of CRC follow the adenoma-carcinoma sequence, with progression from precancerous lesions to malignancy typically taking 5–10 years or even longer (2). Given these factors, CRC is considered one of the most preventable cancers. Early detection and removal of precancerous lesions can interrupt the natural progression of CRC, thereby preventing its development and improving patient survival. In the United States, the natural growth rate of CRC among individuals aged 50–54 and 55–59 years significantly decreased from 1974 to 2014 because of the implementation of screening protocols (3). Furthermore, recent guidelines issued by the American Cancer Society and U.S. Preventive Services Task Force advocates lowering the CRC screening age to 45 years (4, 5). Unlike European countries with high CRC incidence rates, China currently lacks established national guidelines for CRC screening. Additionally, the disparity between the large population and limited medical resources makes it difficult to directly adopt colonoscopy-based screening models used in developed countries. Innovative noninvasive testing methods with high compliance and versatility may be the key to solving this problem.

Colonoscopy remains the gold standard for colorectal neoplasia screening, offering both diagnosis and therapeutic adenoma removal options. However, its invasiveness, complexity, and cost limit compliance rates to 60%, particularly in resource-limited settings (6). Noninvasive alternatives, such as the fecal immunochemical test (FIT), improve participation due to their cost-effectiveness and simplicity (7, 8). However, it lacks sensitivity for non-bleeding lesions and yields high false positives in benign bleeding conditions (9, 10). According to the European Society for Medical Oncology guidelines, elevated carcinoembryonic antigen (CEA) levels are considered a risk factor for recurrence in stage II CRC (11). However, the National Institute of Clinical Biochemistry guidelines do not recommend CEA for population-based early CRC screening because of its insufficient sensitivity and specificity (12). New laboratory testing biomarkers have critical advantages over traditional screening methods. These biomarkers are typically categorized by sample source into blood- and stool-based tests. Previous studies have reported that fecal DNA testing outperforms blood-based tests in detecting adenomas and early stage CRC (13). Furthermore, blood biomarkers are often associated with multiple cancer types, whereas stool samples provide more specific tumor localization, particularly in gastrointestinal cancers (14).

DNA methylation is one of the earliest epigenetic modification pathways discovered (15). Studies have shown that various methylation-based molecular methods have significant potential for detecting colorectal neoplasia with different levels of sensitivity (16, 17). The sensitivity of individual and dual methylation markers for CRC and AA remains limited. To enhance detection sensitivity, methylation markers are frequently combined with other indicators such as gene mutations (18). However, this combination usually prolongs the detection cycle and increases the false-positive rate. Currently, the most sensitive DNA methylation screening test is approved by the U.S. Food and Drug Administration (FDA) is Cologuard^®^ (Exact Sciences), which has reported a sensitivity of 92% for CRC and 42% for AA (19). Here, we validated a fecal DNA test kit using a three-marker methylation panel. Syndecan-2 (SDC2) is instrumental in tumor angiogenesis, invasion, and dissemination through the activation of tumor-derived fibroblasts and their associated signaling pathways (20). The detection of methylated SDC2 in stool samples has demonstrated a sensitivity ranging from 77.0% to 93.9% and specificity between 88.2% and 98.1% (21). The secreted frizzled-related protein 2 (SFRP2) gene encodes glycoproteins that act as inhibitors of the Wnt signaling pathway. Aberrant promoter methylation of SFRP genes has been observed in CRC patients (22, 23). Methylation of SFRP2 has a sensitivity of 77%-90% and specificity of 77% for CRC detection (24). Tissue factor pathway inhibitor (TFPI2) plays a crucial role in safeguarding the extracellular matrix of cancer cells from degradation and inhibiting colony formation and proliferation *in vitro* (25). Loss of TFPI2 function has been associated with increased cellular invasiveness, underscoring its pivotal role in the later stages of carcinogenesis (26).

Based on previous studies and the existing literature, SDC2, SFRP2, and TFPI2 have been proposed to possess diagnostic potential for colorectal neoplasia. Our study assessed the clinical efficacy of combined SDC2/SFRP2/TFPI2 methylation in CRC and AA, comparing its diagnostic performance with that of FOBT and serum CEA to establish its superiority as an innovative screening strategy.

## 2 Materials and methods

### 2.1 Clinical procedures and participants

**Figure 1:**
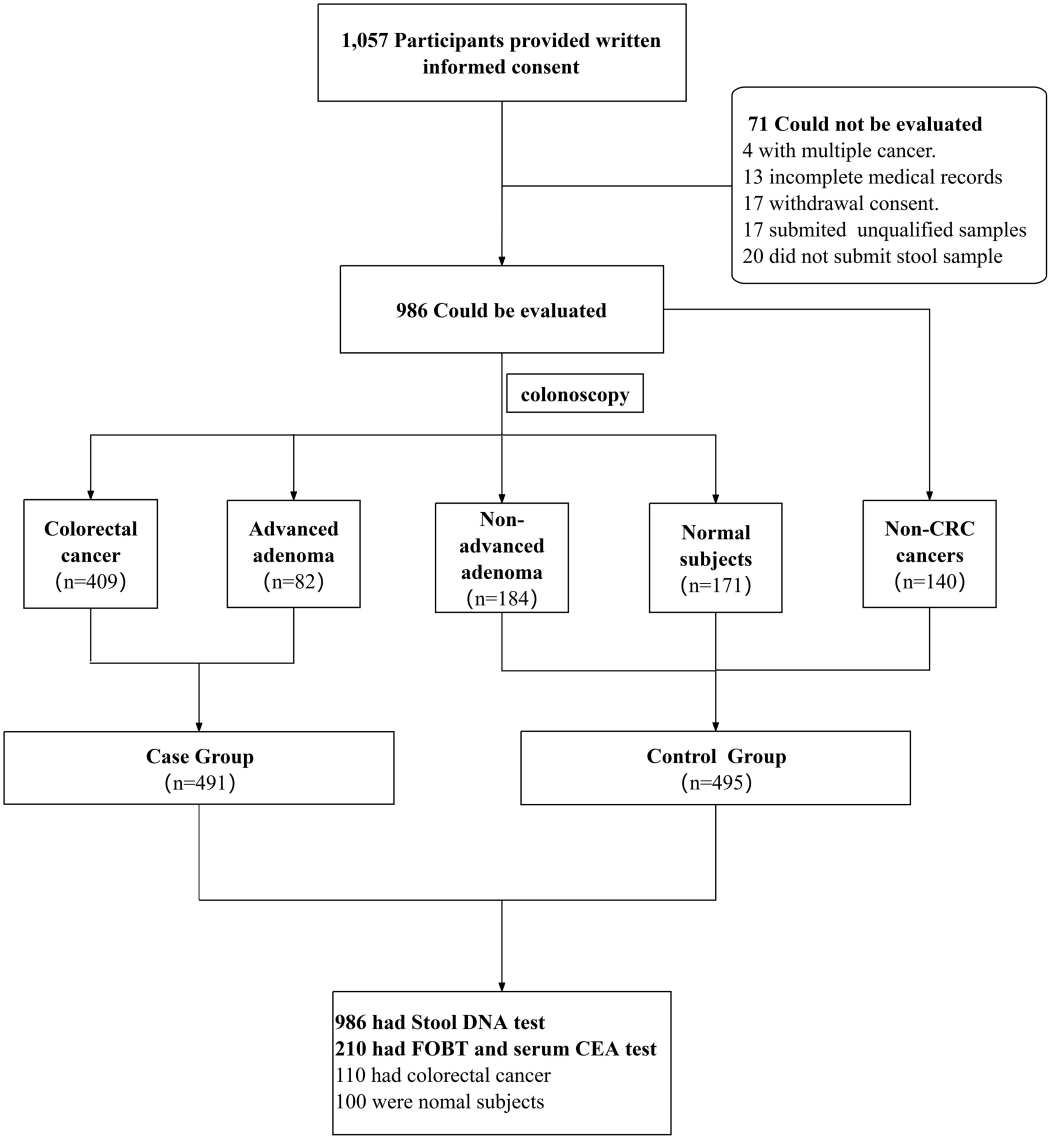
Flow diagram of subject enrollment.

This multicenter case-control study was conducted at four institutions: Tianjin Medical University General Hospital, Shanxi Cancer Hospital, Wenzhou Central Hospital, and Hubei Cancer Hospital. The overall study design is shown in **Figure 1**. The study protocol received approval from the institutional review boards of the participating centers and was registered with the Chinese Clinical Trial Registry (Registration number: ChiCTR2300077757, 2023-11-17). Participants were stratified into five diagnostic groups based on colonoscopic and histopathological findings: (1) CRC, (2) AA, (3) non-AA (including non-AA, non-adenomatous polyps, and other benign intestinal diseases such as enteritis, melanosis coli, and intestinal diverticula), (4) other malignancies (covering nine specified cancer types including breast, ovarian, liver, and gastric cancer), and (5) healthy controls. All the participants provided written informed consent. The inclusion criteria were as follows: (i) age 18–75 years, (ii) no prior history of surgery or chemoradiotherapy, and (iii) either scheduled for a colorectal examination or with a confirmed CRC/AA diagnosis. Pathological verification was also required for subgroups with other malignancies. The exclusion criteria were as follows: (a) concurrent CRC and extra-colonic malignancies, (b) insufficient/inadequate biological samples, and (c) incomplete baseline data (demographics, endoscopic/pathological records).

Between July 2022 and March 2024, 1,057 participants were enrolled in this study, of whom 986 (93.3%) completed the full evaluation. Participants were divided into mutually exclusive groups: 409 patients with CRC, 82 patients with AA, 184 non-AA patients, 171 healthy individuals, and 140 patients with non-CRC cancers. The study procedure for each enrolled participant began with fecal sample collection. All participants provided written informed consent prior to specimen collection. For patients with intestinal diseases, fecal samples (> 5 g) were collected either before surgical resection of intestinal tumors or prior to bowel preparation for colonoscopy. Patients with non-CRC cancers were not required to undergo colonoscopy but were requested to provide stool samples before surgery or treatment. All participants were subsequently divided into case and control groups based on colonoscopy and pathology results. Following specimen collection, quantitative analysis of SDC2, SFRP2, and TFPI2 gene methylation levels was performed on fecal samples from all enrolled participants. Clinical and pathological data were collected from all participants. If multiple lesions were identified in the colon and rectum, only the most advanced lesion was evaluated. A distal lesion was defined as a lesion distal to and including the splenic flexure of the colon.

Additionally, comparative assessments of FOBT and serum CEA levels were conducted in a predefined subgroup comprising 110 treatment-naïve CRC patients and 100 healthy controls recruited from the Department of Clinical Laboratory at Tianjin Medical University General Hospital. Serum CEA levels were measured using an immunoassay conducted with a fully automated chemiluminescence immunoassay analyzer, which operates on the principle of specific antigen-antibody interactions. Serum CEA concentrations of ≥ 5.0 ng/mL were considered positive, while concentrations < 5.0 ng/mL were considered negative. For FOBT, fresh stool samples were analyzed using a clinically approved immunochromatographic assay kit, with results interpreted as positive (+ to +++) or negative (–) based on the color intensity of the test bands on the assay strips.

### 2.2 Laboratory procedures

The stool sample was placed in a sample storage tube (Holosensor Medical Ltd.), thoroughly mixed with a storage solution, and processed within 3 days at room temperature or stored at -80°C. Subsequently, genomic DNA extraction and bisulfite conversion were performed according to the manufacturer instructions. Specifically, 20 µL of the extracted sDNA samples was added to a sterile polymerase chain reaction (PCR) tube, followed by 25 µL bisulfite solution (0.58 mg/µL) and 5 µL hydroquinone. The mixture was vortexed thoroughly and loaded onto a real-time PCR system (ABI QuantStudio™ 5) for bisulfite conversion. Bisulfite conversion was performed using three thermal steps: 98°C for 10 min (denaturation), 64°C for 90 min (conversion), followed by a 4°C hold. Guanidine thiocyanate (4 mol/L), sodium acetate (3 mol/L, pH 5.2), and purified magnetic beads were added, and the mixture was incubated for 15 min. After incubation, the sample was washed (PEG, NaCl, Tris-HCL, and EDTA) and digested (NaOH) twice. Finally, the DNA sample was eluted with nuclease-free water and stored at -20°C. Subsequently, PCR amplification was performed. A total of 14 µL of purified nucleic acid was added to a PCR tube containing a premixed reaction mixture, and the samples were fully mixed and loaded onto a real-time PCR system. The PCR procedure was as follows: initial denaturation at 95°C for 180 s (1 cycle); denaturation at 95°C for 10 s (45 cycles); and annealing, extension, and fluorescence detection at 60°C for 32 s (45 cycles). The thermal cycling parameters were optimized for the ABI QuantStudio™ 5 system, with fluorescence signals collected at the end of each annealing-extension step for the FAM (SDC2), VIC (SFRP2), CY5 (TFPI2), and ROX (ACTB) channels. The transformed DNA fragments of the four genes were detected by quadruple fluorescence PCR amplification: SDC2 (FAM), SFRP2 (VIC), TFPI2 (CY5), and the internal control gene ACTB (ROX). ACTB, which encodes β-actin, is commonly used to assess the content and quality of sample DNA during detection. The gene primers are listed in **Supplementary Table 1**.

Threshold cycles were automatically determined using the QuantStudio™ Design & Analysis Software with the following cutoffs: FAM/VIC/CY5 channels at Ct < 37 and ROX channel at Ct < 40. If the Ct value of ACTB was ≥ 40, the reaction was considered invalid. Methylation positivity for the target gene was defined as an ACTB Ct value < 40 combined with a Ct value < 37 for any one of SDC2, SFRP2, or TFPI2 genes. A negative result was assigned when the FAM/VIC/CY5 channels yielded a Ct value ≥ 37 and/or no detectable Ct value, while the Ct value of the ROX channel was < 40. To normalize variations in DNA input, ΔCt values (ΔCt = Ct(target gene) - Ct(ACTB)) were used to quantify the methylation levels of SDC2, SFRP2, and TFPI2. Lower ΔCt values indicated higher methylation. For samples with undetectable target gene methylation (Ct ≥ 45), ΔCt was calculated as 45–Ct(ACTB). Samples with undetectable ACTB (Ct ≥ 40) were excluded.

### 2.3 TCGA methylation data analysis

DNA methylation profiles of colon adenocarcinoma (TCGA-COAD, n = 443 tumors/45 normals) and rectal adenocarcinoma (TCGA-READ, n = 172 tumors/7 normals) were obtained from the Genomic Data Commons (GDC) data portal using the “TCGAbiolinks” R package (27). After merging the cohorts and applying BMIQ normalization, we analyzed the CpG sites within the TSS1500/TSS200 regions of SDC2, SFRP2, and TFPI2, excluding cross-reactive probes and those with a detection p > 0.01 in >20% of the samples. Gene-level methylation β-values were averaged, and differential methylation was evaluated using paired Wilcoxon signed-rank and unpaired Mann-Whitney U tests. Statistical significance was defined as a Benjamini-Hochberg false discovery rate of < 0.05. All analyses were performed using the R packages “limma” and “minfi.”

### 2.4 Quantification and statistical analysis

Statistical analyses were performed using SPSS Statistics 29.0 (IBM), GraphPad Prism 10.0, and R 4.3.2. Count data are presented as frequencies and percentages, and comparisons between groups were performed using the χ2 test. Sensitivity and specificity for qualitative results were calculated using crosstabs, and logistic regression was used for quantitative results. A logistic regression model was employed to construct the prediction model (**Supplementary Methodology**). Associations between stool DNA results and CRC/AA characteristics were evaluated using the χ2 test or Fisher’s exact test for samples with low expected counts in the contingency table. The diagnostic efficacy for CRC and AA was assessed using ROC curves. Statistical significance was defined as *P < 0.05, **P < 0.01, and ***P < 0.001 (ns: not significant).

## 3 Results

### 3.1 Study population

A total of 986 individuals were comprehensively evaluated and categorized into the case or control group. The case group included 409 patients with CRC and 82 patients with AA (**Table 1**). The control group comprised 495 participants with negative lesions (e.g., non-AA, inflammatory polyps, inflammatory bowel disease), other non-CRC cancers, or no detectable lesions on colonoscopy examinations. Males accounted for 66.19% (325/491) of the case group and 53.94% (267/495) of the control group. Within the control group, males accounted for more than half of each subgroup, except for healthy subjects (39.77%). The mean age (± standard deviation) of the subgroups was as follows: CRC, 61.7 ± 9.3 years; AA, 56.8 ± 11.4 years; non-AA, 55.9 ± 11.9 years; non-CRC cancers, 62.1 ± 8.8 years; and healthy subjects, 48.8 ± 11.8 years. Patients with any type of cancer were, on average, 4.9 years older than those with other conditions. Distal lesions were present in 82.2% (336/409) of patients with CRC and 74.4% (61/82) of patients with AA. Among patients with CRC, 65 had stage I, 107 had stage II, 159 had stage III, and 50 had stage IV disease, with an additional 28 patients lacking staging information. The detailed information is provided in **Table 1**.

**Table 1:** Characteristics of the cases and the controls.

Characteristics	Case group		Control group		
	Colorectal cancer	Advanced colorectal adenoma	Non-advanced adenoma*	Non-CRC cancers	Normal subjects
	409	82	184	140	171
Gender					
Male	267	58	106	93	68
Female	142	24	78	47	103
Age (Mean ± SD)	61.7 ± 9.3	56.8 ± 11.4	55.9 ± 11.9	62.1 ± 8.8	48.8 ± 11.8
Location					
Proximal	68	19	–	–	–
Distal	336	61	–	–	–
Not available	5	2	–	–	–
Stage					
I	65	–	–	–	–
II	107	–	–	–	–
III	159	–	–	–	–
IV	50	–	–	–	–
Not available	28	–	–	–	–

Non-advanced adenoma*: 74 Non-advanced adenoma, 87 benign polyp, 23 benign colorectal diseases.

### 3.2 Methylation levels of SDC2, SFRP2, and TFPI2 in TCGA and participants

**Figure 2:**
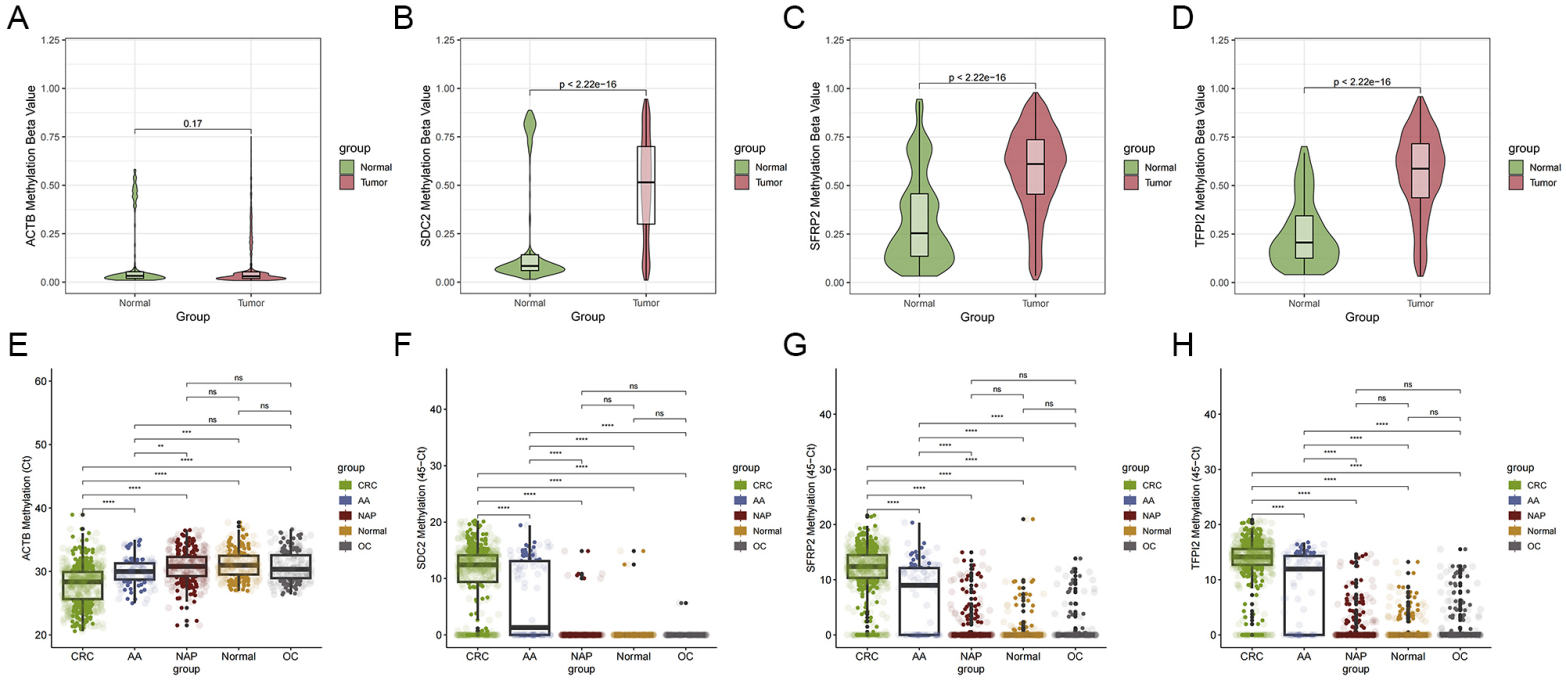
Methylation levels of ACTB, SDC2, SFRP2 and TFPI2.ACTB **(A)**, SDC2 **(B)**, SFRP2 **(C)**, and TFPI2 **(D)** gene methylation levels in CRC and normal tissues from TCGA. Ct values of ACTB **(E)** and ΔCt (45-Ct) values of SDC2 **(F)**, SFRP2 **(G)**, and TFPI2 **(H)** gene methylation in the fecal samples of participants from various groups. CRC, colorectal cancer; AA, advanced adenoma; NAP, non-advanced polyp; OC, other cancer. **P <0.01, ***P <0.001, ****P <0.0001, ns: not significant.

Analysis of TCGA data revealed significantly higher methylation levels of SDC2, SFRP2, and TFPI2 in CRC tissues than in normal controls (P < 0.001), while the internal reference gene ACTB showed no differential methylation (P = 0.17) (**Figures 2A–D**). The average Ct value of ACTB in CRC and AA samples was significantly lower than that in the control samples (**Figure 2E**). Lower ACTB Ct values suggest higher human DNA content in the stool, potentially reflecting increased epithelial shedding. A significant difference was observed in the average 45 - Ct values for SDC2, SFRP2, and TFPI2 between individuals with CRC/AA and those in the other subgroups (P < 0.001, **Figures 2F–H**). Additionally, the methylation levels of the three genes did not differ significantly among the non-advanced polyp, other cancer, and healthy groups. These findings indicate that the methylation levels of these three genes are notably increased in the feces of patients with CRC and AA, demonstrating their potential as diagnostic markers. Among these genes, TFPI2 had the highest methylation levels in the stool samples of patients with colorectal neoplasia, surpassing those of SDC2 and SFRP2.

### 3.3 Analytical performance of the stool DNA test

The stool DNA test successfully detected CRC in 385 of 409 patients, yielding a sensitivity of 94.13% (95% CI: 91.42-96.03) and a specificity of 94.74% in 171 healthy controls (**Table 2**). For AA, the test detected 40 of 82 cases, achieving a sensitivity of 59.76% (95% CI: 48.94-69.70), whereas the detection rates for non-AA were low. For individuals without AA, with benign polyps, and with colorectal disease, the specificity of the test was 91.85% (95% CI: 86.99-95.00) (**Table 2**). When non-AA patients were considered the case group and the remaining patients the control group, specificity was lower in the case group, indicating a greater likelihood of false-positive results for non-AA detection. The remaining patients refers to all control groups in this study, which exclude non-AA and include healthy individuals, benign polyps, benign colorectal diseases, and non-CRC cancers. Concurrently, for individuals with cancer, a slight difference in specificity was observed between non-CRC digestive and non-digestive system cancers (87.76% vs. 95.24%, P = 0.229).

**Table 2:** Sensitivity and specificity of SDC2/SFRP2/TFPI2 panel.

Study group	Patients no.	SDC2		SFRP2		TFPI2		SDC2/SFRP2/TFPI2	
		True positive	Sensitivity% (95% CI)	True positive	Sensitivity% (95% CI)	True positive	Sensitivity% (95% CI)	True positive	Sensitivity% (95% CI)
Colorectal cancer	409	316	77.26 (73.08-81.09)	350	85.57 (81.95-88.74)	363	88.75 (85.39-91.56)	385	94.13 (91.42-96.03)
Stage I	65	55	84.62 (73.78-92.11)	58	89.23 (79.65-95.27)	59	90.77 (81.65-96.23)	61	93.85 (85.22-97.58)
Stage II	107	82	76.64 (67.75-83.99)	90	84.11 (76.02-90.34)	92	85.98 (78.12-91.83)	99	92.52 (85.94-96.16)
Stage III	159	121	76.10 (68.52-82.63)	138	86.80 (80.51-91.78)	145	91.19 (85.82-94.93)	153	96.23 (92.01-98.26)
Stage IV	50	40	80.00 (66.30-89.90)	44	88.00 (75.73-95.48)	45	90.00 (78.16-96.64)	49	98.00 (89.50-99.90)
unknown	28	18	64.29 (43.85-81.38)	20	71.43 (52.09-86.45)	22	78.57 (59.81-91.27)	23	82.14 (64.41-92.12)
Advanced adenoma	82	40	48.78 (37.80-59.90)	45	54.88 (43.70-65.75)	46	56.10 (44.88-66.92)	49	59.76 (48.94-69.70)
Control group	Patients No.	Positive	Specificity% (95% CI)	Positive	Specificity% (95% CI)	Positive	Specificity% (95% CI)	Positive	Specificity% (95% CI)
Normal controls	171	2	98.83 (96.05-99.85)	6	96.49 (93.05-98.43)	4	97.66 (94.68-99.18)	9	94.74 (90.30-97.21)
None advanced controls*	184	4	97.83 (94.89-99.27)	12	93.48 (89.06-96.45)	10	94.57 (90.40-97.32)	15	91.85 (86.99-95.00)
Non-advanced adenom	74	3	95.95 (89.17-98.93)	7	90.54 (82.01-95.87)	6	91.89 (83.65-96.78)	10	86.49 (76.88-92.49)
Benign polyp	87	0	100.00 (96.08-100.00)	3	96.55 (90.74-98.98)	1	98.85 (94.29-99.97)	4	95.40 (88.77-98.20)
Non-CRC cancers	140	0	100.00 (97.37-100.00)	8	94.29 (89.05-97.48)	12	91.43 (85.63-95.45)	14	90.00 (83.91-93.95)
Non-digestive cancers	42	0	100.00 (91.02-100.00)	0	100.00 (91.02-100.00)	2	95.24 (83.81-99.03)	2	95.24 (83.81-99.03)
Non-CRC digestive cancers	98	0	100.00 (96.15-100.00)	8	91.48 (84.59-96.48)	10	89.80 (82.01-95.08)	12	87.76 (79.81-92.85)

None advanced controls*: Non-advanced adenom, benign polyp, benign colorectal diseases.

**Figure 3:**
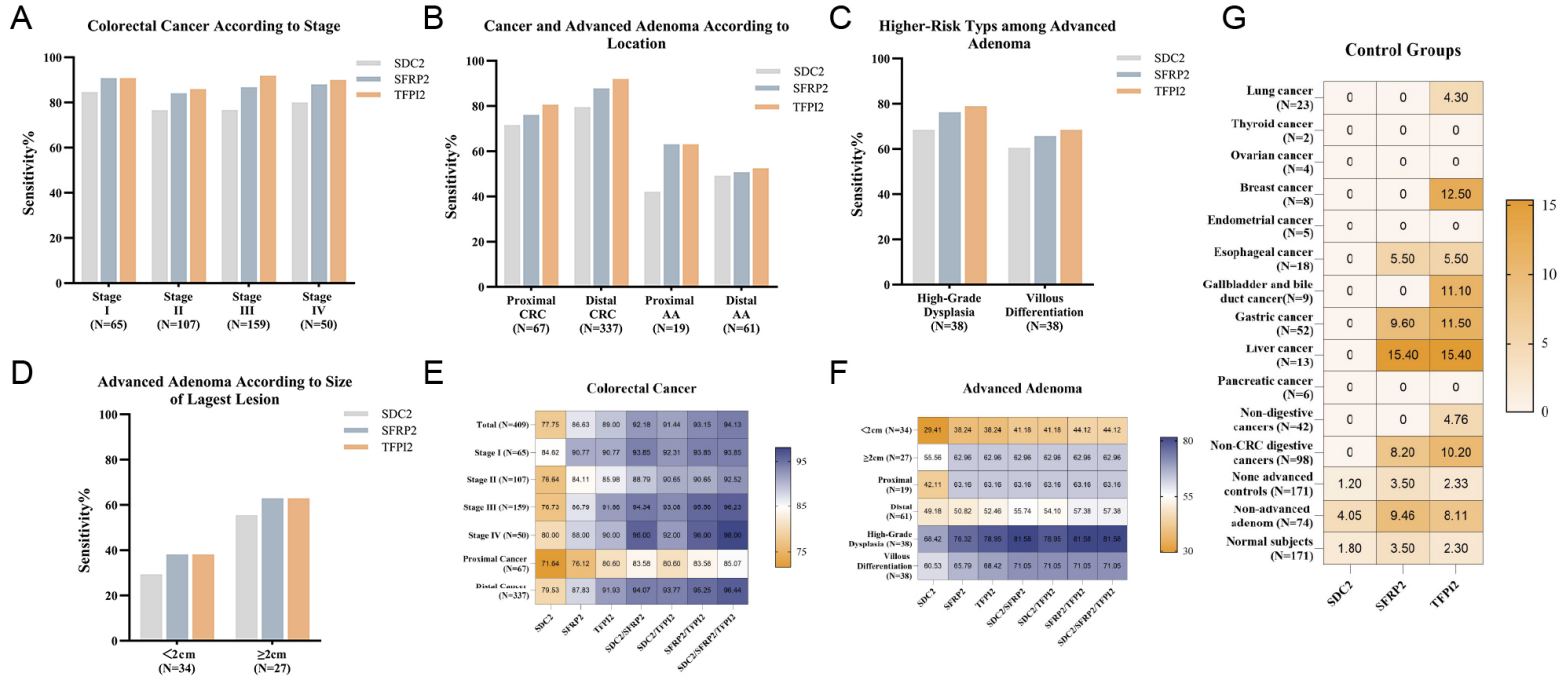
Diagnostic performance of the stool DNA test in colorectal neoplasia detection and specificity analysis. **(A)** Stage-specific sensitivity of SDC2, SFRP2, and TFPI2 for CRC detection. **(B)** Location -dependent sensitivity for CRC and AA detection. **(C, D)** Sensitivities of SDC2, SFRP2 and TFPI2 for the detection of AA according to higher-risk type and lesion size. **(E, F)** Heat map of sensitivities (%) of the stool DNA test for detecting CRC and AA. **(G)** False-positive rates (%) of SDC2, SFRP2, and TFPI2 in control groups.

Subsequently, we performed a sensitivity analysis of the three methylation markers of colorectal neoplasia. Within the CRC group, the sensitivity of combined SDC2/SFRP2/TFPI2 methylation detection for stage I/II CRC was not significantly different from that for stage III/IV CRC (P = 0.102, **Table 3**). Sensitivity increased with advancing CRC stage, except for stage I (**Figure 3A**). Furthermore, the sensitivity for detecting distal CRC was significantly higher than that for detecting proximal lesions (P < 0.05) (**Figure 3B**). Within the AA group, the stool DNA test showed higher sensitivity in patients with high-grade dysplasia or villous differentiation than in those with other AA types (**Figure 3C**). However, sensitivity did not vary significantly according to adenoma size or location (P > 0.05) (**Table 4**, **Figure 3D**). Among the three genes, SDC2 exhibited relatively lower sensitivity across various stages and locations of CRC than the other two genes, whereas TFPI2 demonstrated the greatest sensitivity (**Table 2**, **Figure 3E**). Furthermore, comprehensive genetic panels encompassing multiple genes significantly outperformed single-gene assays in terms of their sensitivity. However, the benefit of combined testing was not particularly pronounced for AAs (**Table 2**, **Figure 3F**). In the control group, SDC2exhibited the highest specificity of the three genes (**Table 2**). Among non-CRC digestive system cancers, SFRP2 and TFPI2 showed false-positive rates of 8.2% (10/98) and 10.2% (8/98), respectively, whereas SDC2 demonstrated no false positives (0%) (**Figure 3G**). In non-digestive system cancers, TFPI2 also produced false positives, notably in lung cancer (1/28) and breast cancer (1/13).

**Table 3:** Stool DNA test results in relation to different tumor characteristics.

Characteristics	SDC2			SFRP2			TFPI2			Combined		
	Negative	Positive	P value	Negative	Positive	P value	Negative	Positive	P value	Negative	Positive	P value
Location (n=404)												
Proximal	20	47	0.103	16	51	0.015*	14	53	0.004*	10	57	<0.0001*
Distal	70	267		42	295		30	307		13	324	
Size (n=226)												
<5cm	32	107	0.500	21	118	0.917	13	126	0.645	9	130	0.809
≥5cm	24	65		13	76		10	79		5	83	
Histology (n=355)												
Adenocarcinoma	62	245	0.277	43	264	0.217	31	276	0.176	16	291	0.383
Mucinous adenocarcinoma	13	35		10	38		8	40		4	44	
Morphology (n=204)												
Protruded type	13	29	0.276	9	33	0.133	7	35	0.093	6	36	0.032*
Ulcerative type	37	125		20	142		13	149		6	156	
Degree of differentiation (n=343)												
High	5	8	0.286	2	11	0.635	4	9	0.041*	2	11	0.131
Medium	52	202		30	224		24	230		12	242	
Poor	15	61		12	64		5	71		2	74	
TNM Stage (n=381)												
I-II	35	137	0.538	24	148	0.768	21	151	0.323	12	160	0.106
III-IV	48	161		27	182		19	190		7	202	
T (n=348)												
Tis-T2	14	66	0.348	13	67	0.468	10	70	0.537	8	72	0.042*
T3-T4	60	208		35	233		27	241		11	257	
N (n=354)												
N0	35	140	0.361	29	146	0.191	23	152	0.148	14	161	0.058
N1-2	43	136		21	158		15	164		6	173	

*P in bold : P< 0.05.

P values were calculated using the x2 test or Fisher exact test in case of low sample number per cell.

**Table 4:** Stool DNA test results in relation to different advanced adenoma characteristics.

Characteristics	SDC2			SFRP2			TFPI2			Combined		
	Negative	Positive	P value	Negative	Positive	P value	Negative	Positive	P value	Negative	Positive	P value
Location (n=80)												
Proximal	11	8	0.590	7	12	0.346	7	12	0.413	7	12	0.655
Distal	31	30		30	31		29	32		26	35	
Size (n=61)												
<20mm	24	10	0.066	21	13	0.055	21	13	0.055	19	15	0.143
≥20mm	12	15		10	17		10	17		10	17	
Histology (n=75)												
Tubular	17	14	0.490	14	17	0.420	15	16	0.420	13	18	0.597
Tubulovillous	15	22		13	24		12	25		11	26	
Hyperplastic	3	4		4	3		3	4		3	4	
Morphology (n=39)												
Flat	2	3	0.080	1	4	0.088	1	4	0.027*	1	4	0.072
Pedunculated	11	3		10	4		11	3		10	4	
Sessile	8	12		8	12		8	12		7	13	
Dysplasia (n=68)												
Low grade	16	9	0.013*	15	10	0.005*	16	9	0.002*	15	10	0.001*
High grade	13	27		10	30		10	30		8	32	

*P in bold :P<0.05.

P values were calculated using the x2 test or Fisher exact test in case of low sample number per cell.

**Figure 4:**
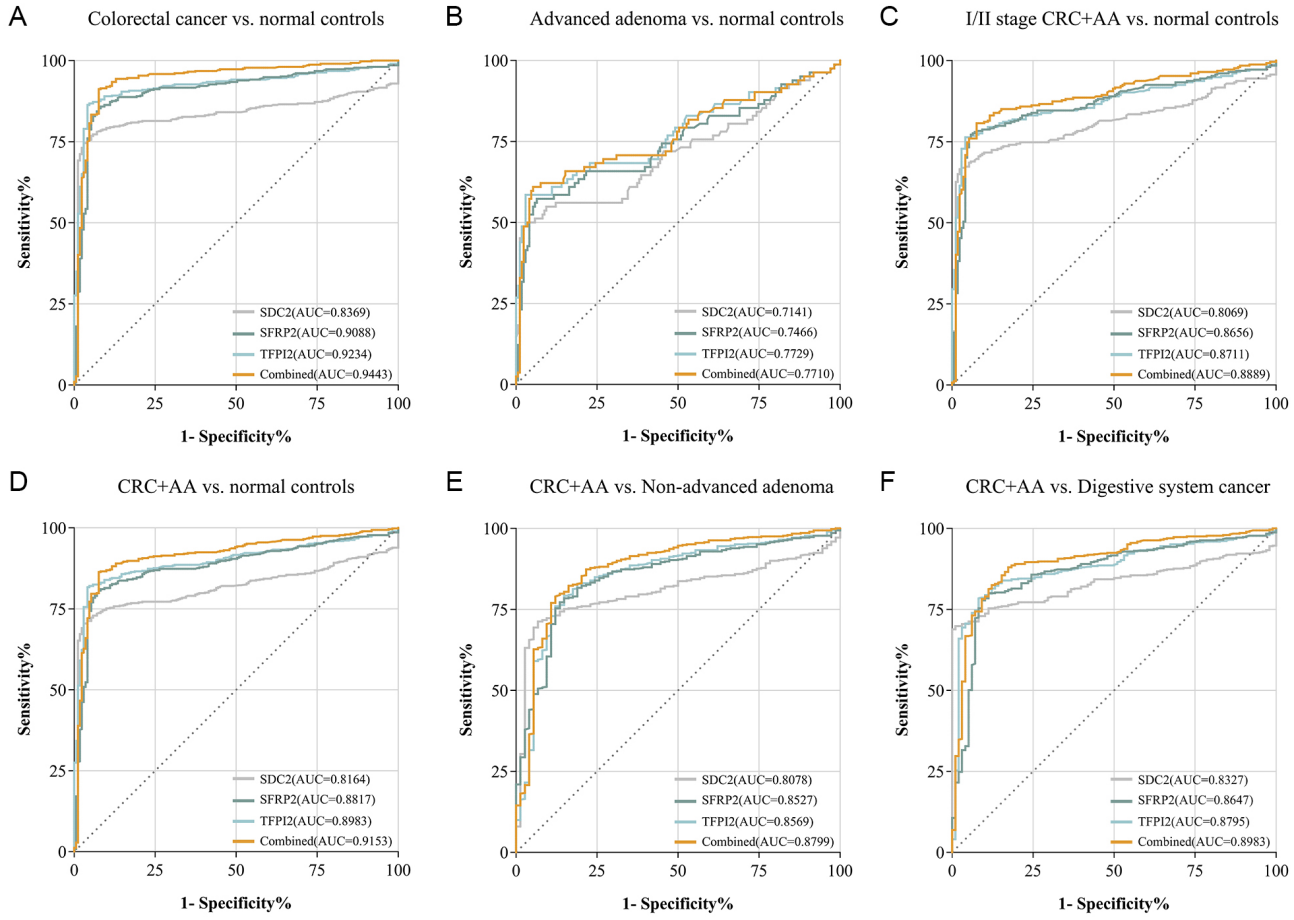
Diagnostic efficacy of the stool DNA test. ROC curves and AUC values were plotted for CRC vs. NC **(A)**, AA vs. NC **(B)**, stage I/II CRC plus AA vs. NC **(C)**, CRC plus AA vs. NC **(D)**, CRC plus AA vs. NAA **(E)**, and CRC plus AA vs. digestive system cancer **(F)**.

The ROC curve for each group was plotted using the ΔCt value. The area under the ROC curve (AUC) with a 95% CI was calculated. ROC curves were generated to evaluate the performance of distinguishing patients with CRC and AA from the control groups. The combination of SDC2/SFRP2/TFPI2 detection accurately identified CRC with an AUC of 0.9343 and AA with an AUC of 0.7729 (**Figures 4A, B**). The AUC of this panel for the diagnosis of early CRC and AA is 0.8889 (**Figure 4C**). The AUC was 0.9153 for patients with advanced colorectal neoplasia (CRC plus AA) compared to healthy controls (**Figure 4D**). Compared with non-AA, the AUC for colorectal neoplasia was 0.8799, and compared with digestive system cancers, the AUC was 0.8983 (**Figures 4E, F**). Among the three genes, TFPI2 demonstrated superior discrimination ability between the CRC and control groups, as measured by AUC. In addition, the combined performance of SDC2, SFRP2, and TFPI2 surpassed that of individual genes. Interestingly, TFPI2 alone achieved an AUC of 0.7729 for AA detection, which was higher than that of the combined panel (AUC = 0.7710). Notably, a decrease in AUC values was observed for SFRP2, TFPI2, and the combination of all three markers when distinguishing CRC patients from healthy individuals compared with differentiating CRC patients from those with digestive system cancers. Conversely, the AUC value for SDC2 improved, increasing from 0.8078 to 0.8327 after the fine-tuning.

### 3.4 Stool DNA test results in relation to colorectal neoplasia characteristics

We investigated the clinicopathological characteristics of CRC and AA patients and assessed the positive identification rates of the three genes individually and in combination. The locations of the CRC lesions were recorded. SFRP2, TFPI2, and the combination of gene panel achieved higher positive detection rates in individuals with distal lesions than in those with proximal lesions (P = 0.015, P = 0.004, P < 0.0001) (**Table 3**). In addition, a positive result from the combined panel was associated with the presence of ulcerative lesions (P = 0.032) and tumor infiltration depth (P = 0.042). In 204 CRC cases, where the degree of differentiation was recorded, an increase in positive results for TFPI2 was observed as the degree of differentiation decreased (P = 0.041). However, no statistically significant differences were observed in tumor size, morphology, TNM stage, or N stage. Importantly, the positive SDC2 expression rate did not correlate with any tumor characteristics. For individuals with AA, stool DNA test results were evaluated for associations with lesion location, size, histology, morphology, and degree of dysplasia. Both individual markers and the combined panel were more frequently positive in patients with AA exhibiting high-grade dysplasia (**Table 4**). Additionally, the positive rate of TFPI2 was associated with lesion morphology, with detection rates of 80.0% (4/5) for flat lesions and 21.4% (3/14) for pedunculated lesions. Interestingly, all flat polyps identified as positive were laterally spreading.

### 3.5 Performance of the stool DNA test compared with FOBT and serum CEA

We subsequently compared the stool DNA assay with conventional, noninvasive screening methods. FOBT and serum CEA tests were performed in 210 participants, including 110 patients with CRC and 100 healthy controls (**Table 5**). The stool DNA test demonstrated significantly higher sensitivity (88.18% vs. 73.65%) and specificity (98.00% vs. 77.00%) than FOBT. Notably, the stool DNA test outperformed the serum CEA test across all CRC stages, with a particularly pronounced difference in stage I disease (85.71% vs. 0% sensitivity, respectively). The stool DNA test also showed superior performance compared with FOBT, with sensitivities of 85.71% vs. 57.14% for stage I CRC and 88.46% vs. 69.23% for stage II CRC, respectively.

**Table 5:** Sensitivity and specificity of the SDC2/SFRP2/TFPI2 panel, FOBT, and serum CEA level.

Study group	Patients no.	SDC2/SFRP2/TFPI2		FOBT		CEA	
		Positive	Sensitivity% (95% CI)	Positive	Sensitivity% (95% CI)	Positive	Sensitivity% (95% CI)
Colorectal cancer	110	97	88.18 (80.82-92.96)	81	73.64 (64.71-80.97)	48	43.64 (34.74-52.96)
Stage I	14	12	85.71 (57.14-97.06)	8	57.14 (32.59-78.62)	0	0.00 (0.00-21.53)
Stage II	26	23	88.46 (70.09-97.41)	18	69.23 (50.01-83.50)	12	46.15 (28.76-64.54)
Stage III	48	42	87.50 (74.71-95.35)	36	75.00 (61.22-85.08)	23	47.92 (34.47-61.67)
Stage IV	15	14	93.33 (68.05-99.78)	15	100 (79.61-100.0)	9	60.00 (35.75-80.18)
unknown	7	6	85.71 (48.68-97.47)	4	57.14 (25.05-84.18)	4	57.14 (25.05-84.18)
Normal controls*	100	2	98.00 (93.00-99.64)	23	77.00 (67.85-84.16)	9	91.00 (83.77-95.19)

Normal controls* refers to the control population. Values in the "Positive" column represent false positive counts, and values in the "Sensitivity (95% CI)" column are actually specificity.

**Figure 5:**
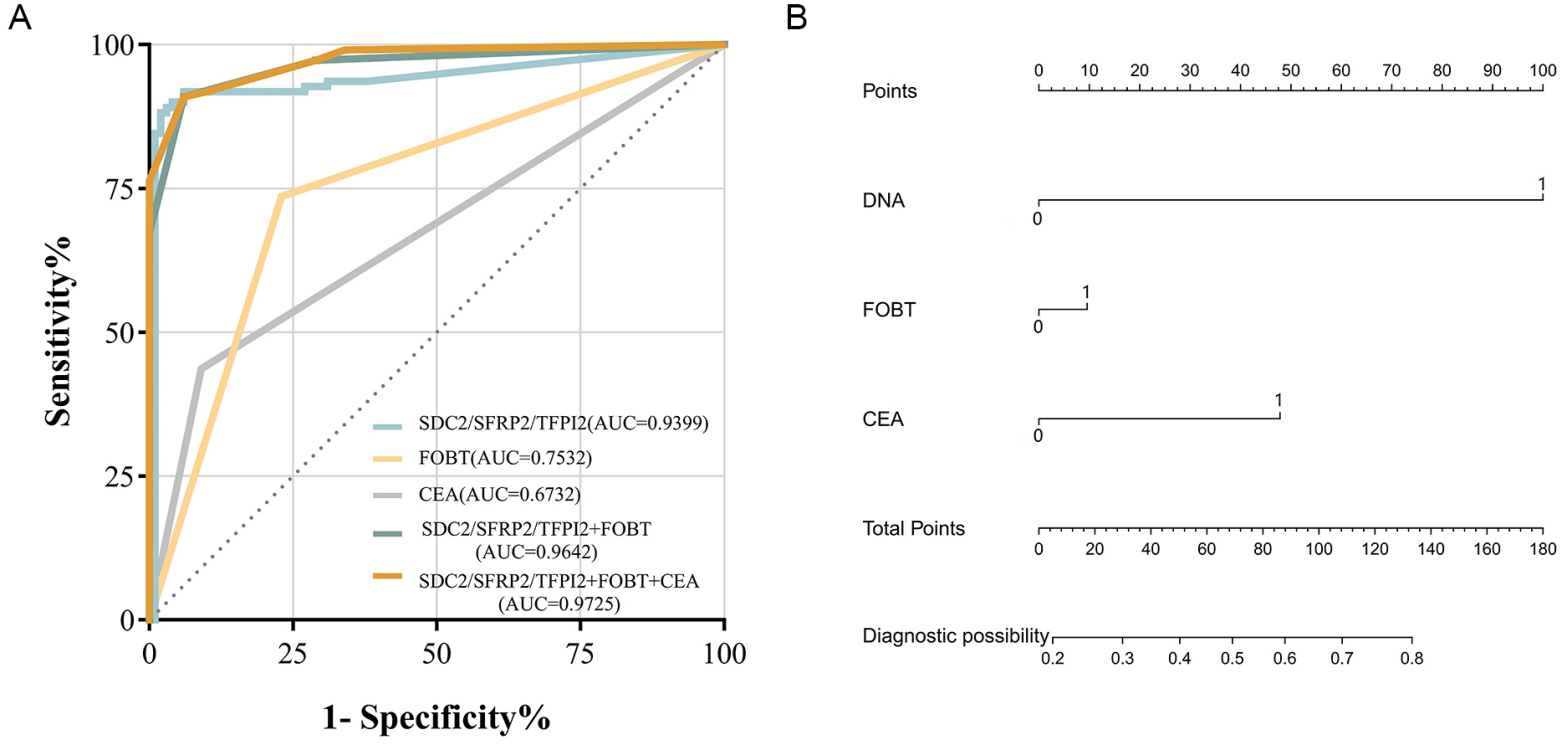
Comparative diagnostic efficacy of SDC2/SFRP2/TFPI2, FOBT, and CEA for CRC detection. **(A)** Efficacy of fecal SDC2/SFRP2/TFPI2, FOBT, and CEA. **(B)** Nomogram-based prediction model for the risk of developing CRC using logistic regression.

This study integrated stool DNA testing with conventional detection methods and employed logistic regression to independently construct predictive models for the stool DNA test, FOBT, and CEA, as well as a combination of all three assays. ROC curve analysis was used to ascertain the AUC (**Table 6**, **Figure 5A**). SDC2/SFRP2/TFPI2 methylation detection combined with FOBT achieved a sensitivity of 67.27%, specificity of 99.01%, and AUC of 0.9642. Furthermore, the detection efficacy of SDC2/SFRP2/TFPI2 combined with FOBT and serum CEA results further improved performance, with sensitivity, specificity, and AUC values of 90.91%, 94.00%, and 97.25%, respectively. The coefficients for the SDC2/SFRP2/TFPI2, FOBT, and CEA methods individually were 5.221, 2.514, and 1.693, respectively, while the model’s constant term had a coefficient of -3.887 in this model. To assist clinicians, we created nomograms to estimate individual CRC risk probabilities based on multimodal data (**Figure 5B**). Additionally, we evaluated an alternative approach for defining the composite result, wherein a positive outcome in any single test was considered a composite-positive result. Our study revealed that combining the stool DNA test with FOBT and CEA identified more CRC cases, achieving a combined sensitivity of 99.09%. However, this approach reduced specificity to 66.6% (95% CI: 56.3-74.5), leading to a higher rate of false positives (**Table 6**). Overall, compared to individual detection methods, the combined approach significantly improved the positive detection rates for CRC, suggesting that the three methods are complementary and can identify more CRC cases when used together.

**Table 6:** Efficacy of fecal SDC2/SFRP2/TFPI2, FOBT, and serum CEA for CRC diagnosis.

Detection method	Sensitivity% (95% CI)	Specificity% (95% CI)	AUC(95% CI)	P value
SDC2/SFRP2/TFPI2	0.8818 (0.8082-0.9296)	0.9800 (0.9300-0.9964)	0.9399 (0.9019-0.9779)	<0.0001
FOBT	0.7364 (0.6471-0.8097)	0.7700 (0.6785-0.8416)	0.7532 (0.6857-0.8207)	<0.0001
CEA	0.4364 (0.3474-0.5296)	0.9100 (0.8377-0.9519)	0.6732 (0.6003-0.7460)	<0.0001
SDC2/SFRP2/TFPI2 +FOBT*	0.6727 (0.5805-0.7533)	0.9901 (0.9460-0.9995)	0.9642 (0.9399-0.9884)	<0.0001
SDC2/SFRP2/TFPI2 +FOBT+CEA*	0.9091 (0.8407-0.9499)	0.9400 (0.8752-0.9722)	0.9725 (0.9536-0.9913)	<0.0001
SDC2/SFRP2/TFPI2 +FOBT+	0.9727 (0.9229-0.9926)	0.7100 (0.6146-0.7899)	0.8414 (0.7832-0.8995)	<0.0001
SDC2/SFRP2/TFPI2 +FOBT+CEA+	0.9909 (0.9503-0.9995)	0.6660 (0.5628-0.7454)	0.8255 (0.7649-0.8860)	<0.0001

*Use logistic regression to build prediction curves and ROC curve analysis to calculate the area under the curve.

+Result was considered positive if any one of them has a positive result.

## 4 Discussion

Compared to normal cells, tumor cells exhibit accelerated proliferation due to genomic instability and reduced adhesion to the basement membrane, facilitating enhanced shedding into the intestinal lumen (28). Unlike normal apoptotic cells, tumor-derived DNA resists degradation through mechanisms such as defective apoptosis and necrosis, persisting in the fecal matrix. The intestinal microenvironment, along with stool stabilizers, facilitates the preservation of tumor-derived DNA in fecal samples. These features make fecal samples ideal for colorectal neoplasia screening using epigenetic biomarkers. Cologuard detects methylated BMP3/NDRG4, mutant KRAS, and FIT (18). Building on this approach, China-developed assays such as ColoClear™ have achieved 91.9% CRC sensitivity and 63.5% AA sensitivity in high-risk populations (29). Despite these screening methods being effective, their high cost remains a barrier that requires price-reduction strategies for global implementation (30). Studies suggest that DNA hypermethylation at promoter regions is an effective biomarker for early carcinogenesis. Although individual methylation markers have demonstrated diagnostic potential (31, 32), their clinical utility is often limited by tumor heterogeneity and molecular diversity. To enhance their accuracy, these markers are typically combined with protein biomarkers or somatic mutation analyses. To overcome the limitations of single-marker approaches, we developed a novel tri-marker assay targeting SDC2/SFRP2/TFPI2 methylation, which achieved 94.13% sensitivity for CRC detection and 59.76% sensitivity for AA, with 94.74% specificity in healthy controls.

Its performance surpasses that of the published multi-methylation assays. For example, a study in 2023 evaluating the efficacy of SDC2/ADHFE1/PPP2R5C panel in CRC detection reported a sensitivity of 84.8%, a specificity of 98.0%, and an AUC of 0.930 (95% CI: 88.9–97.0) (33). A 2024 study by another research team reported that the diagnostic sensitivity of the combination of SDC2 and NDRG4 methylation for CRC was 84.8%, whereas the specificity was 90% (22). A dual-target stool DNA test that detects methylated SDC2 and TFPI2 exhibited higher sensitivity for CRC than either marker individually, although its specificity decreased (34). Similar trends were observed in this study; the sensitivity of the combined assay for CRC was 94.13%, which was greater than the values of 77.26% for SDC2, 85.57% for SFRP2, and 88.75% for TFPI2 alone, and the specificity in healthy controls was 94.74%, which was lower than the values of 98.83% for SDC2, 96.49% for SFRP2, and 97.66% for TFPI2 alone. Subsequently, we assessed the significance of each indicator individually and in combination by employing logistic regression and ROC curve analyses. The AUC considers both true-positive and false-positive rates, enabling a more thorough evaluation of the prediction efficacy of a model. The combined detection curve exhibited an AUC of 0.9153 for colorectal neoplasia, whereas the AUCs for the individual indicators SDC2, SFRP2, and TFPI2 were 0.8155, 0.8817, and 0.8983, respectively. Despite the potential compromise in specificity associated with combination testing, our data indicate that the combined assay achieved the greatest AUC, yielding the most effective detection.

A pivotal strength of this study lies in the inclusion of 140 non-CRC cases, which allowed us to rigorously evaluate the specificity of the assay. It demonstrated 90.00% specificity in distinguishing CRC from other malignancies, with false positives predominantly driven by SFRP2 and TFPI2 methylation in digestive system cancer. This observation aligns with prior reports of methylation of these genes in gastrointestinal malignancies (35–37), where SFRP2 acts through Wnt signaling and TFPI2 via extracellular matrix (ECM) remodeling. Importantly, SDC2 methylation was absent in all non-CRC digestive cancers, confirming its CRC specificity. This dichotomy highlights SFRP2/TFPI2 as pan-gastrointestinal cancer markers and SDC2 as a CRC-selective biomarker. Additionally, the false-positive rates of SDC2, SFRP2, and TFPI2 in the control group were 1.21%, 5.25%, and 5.25%, respectively. The increased sensitivity of SFRP2 and TFPI2, along with the increased specificity of SDC2, indicates that these biomarkers may play complementary roles in CRC diagnosis. There is often a trade-off between sensitivity and specificity, with high sensitivity potentially corresponding to diminished specificity and vice versa. Therefore, it is unlikely that a single biomarker could possess the ideal combination of sensitivity and specificity, highlighting the necessity of using multiple markers in combination. The benefit of combined markers in the diagnosis of AA was less significant than that of CRC. The AUC values for TFPI2, SFRP2, and SDC2 were 0.7729, 0.7466, and 0.7131, respectively, and the AUC for the combination was 0.7710. Methylation is less prevalent in adenomatous tissues than in CRC. Additionally, adenomas may exhibit greater heterogeneity, indicating that methylation patterns may differ among adenomas. We hypothesized that TFPI2 would demonstrate a higher AUC than the tri-marker panel because of its increased susceptibility to methylation during the early adenoma-carcinoma transition, making it the most sensitive marker for AA detection. In contrast, SDC2 and SFRP2 exhibited low methylation rates in adenomas. The inclusion of SDC2 and SFRP2 did not significantly enhance the detection rate of AA; instead, the contribution of TFPI2 was diminished owing to the low sensitivity of these two markers.

Given the high heterogeneity of CRC, the methylation status of a single target may vary across different subtypes of the disease, potentially impairing the detection performance. In a study examining CRC methylation groups defined by SDC2 and TFPI2, the authors reported higher methylation levels of TFPI2 in left-sided tumors and SDC2 in right-sided tumors (38). Among the three genes, SDC2 showed the highest sensitivity for proximal CRC. In contrast, SFRP2, TFPI2, and the combined assay demonstrated better performance in detecting distal CRC. One potential explanation is that SDC2, SFRP2, and TFPI2 may exhibit distinct methylation patterns in different CRC regions. Another possible reason is that tumor cells shed from proximal CRC lesions undergo extensive decomposition and destruction. We found that TFPI2 alone demonstrated a significant ability to detect poorly differentiated CRC, suggesting that abnormal TFPI2 gene methylation may be associated with the malignant biological behavior of tumors and could serve as a marker of poor prognosis. Additionally, our results show that combined testing exhibits an enhanced capability for detecting T3-T4 stage CRC. At this stage, CRC typically expands to deeper layers of the intestinal wall and may even penetrate it, leading to a greater rate of cell necrosis and shedding. Ultimately, more cancer cells and their DNA are released into the intestinal lumen of the host. Additionally, T3-T4 stage tumors display more aggressive biological behavior and typically exhibit more pronounced molecular characteristics and genetic alterations, which may be associated with greater levels of abnormal methylation (39). The high detection rate of advanced tumors using these biomarkers suggests their greater sensitivity in detecting more advanced CRC; therefore, these markers may be more suitable for screening or diagnosing late-stage CRC and may serve as potential indicators for prognostic assessment of CRC.

In patients with AA and severe dysplasia, the methylation of related genes is more prevalent and pronounced. Severe dysplasia, a type of precancerous lesion, exhibits cellular morphology and tissue structures similar to those of CRC cells, indicating a higher malignant potential (40). Gene methylation likely occurs early in the progression of adenomas to cancer, which may explain the higher detection rate in high-risk adenomas. Consistent with previous findings (41), high-grade dysplasia was detected with greater sensitivity in the AA group. Notably, TFPI2 had a significantly greater sensitivity for flat AAs than for non-flat AAs. All flat AAs identified as positive were laterally spreading tumors (LSTs). These tumors typically remain flat or grow laterally rather than protruding into the intestinal lumen (42). This distinct growth pattern makes LSTs more challenging to detect during endoscopic examinations, thereby increasing the risk of misdiagnosis or missed diagnoses. Therefore, biomarkers such as TFPI2, which shows a higher frequency of methylation in adenomas, may be particularly valuable for their detection.

The major challenges of preliminary screening using an adopted risk score or FOBT in countries with large populations but limited colonoscopy resources are the high positive rate and low detection rate. A recent nationwide screening program identified 13% of 1.3 million participants as high-risk CRC individuals; however, colonoscopy compliance among these patients was only 15% (43). Although repeated FIT has been attempted to improve detection rates and effectiveness, its success remains limited (44). In this study, we evaluated the diagnostic effectiveness of combining SDC2/SFRP2/TFPI2 methylation with FOBT and CEA levels. The combined detection method achieved the highest AUC of 0.9725 for CRC, significantly outperforming FOBT and CEA. However, a simpler approach that considers any positive result among the three indicators as a positive sample led to an increase in false positives, primarily due to the lower specificity of FOBT. Specificity is also important because it directly affects the number of individuals referred for unnecessary colonoscopies. While our results suggest that combining the three methods maximizes detection efficacy, practical considerations, such as cost-effectiveness and population compliance, must be addressed to develop a feasible screening strategy.

This study highlights the significant advantages of a novel methylation panel, which simplifies detection and mitigates technical challenges by obviating the need for concurrent gene mutation or protein biomarker detection, unlike other assays such as Cologuard. This streamlined methodology contributes to cost reduction in the process. Furthermore, while most fecal test kits face a trade-off between sensitivity and specificity, the SDC2/SFRP2/TFPI2 panel achieves both high sensitivity and specificity owing to the complementary functions of its markers. This resulted in enhanced detection of early stage CRC and precancerous lesions while maintaining high specificity against other diseases. Our tri-marker panel comprehensively addresses the entire spectrum of colorectal carcinogenesis, offering a stratified diagnostic utility. Unlike most assays that primarily focus on CRC detection, this study provides extensive data on AA, non-AA, benign polyps, non-CRC cancers, and healthy individuals. Notably, we demonstrated that the stool DNA test can effectively differentiate CRC from non-CRC digestive cancers, thereby reducing the number of patients with other digestive malignancies who undergo unnecessary colonoscopies. However, this study has some limitations. Large-scale validation is required to accurately assess the effectiveness of the combined detection strategy. Due to the insufficient representation of advanced serrated polyps with hypermethylation phenotypes, the sensitivity for non-AA precursor lesions may be underestimated (45). Furthermore, the mean age difference between healthy controls (48.8 years) and CRC patients (61.7 years) may introduce bias, potentially resulting in an overestimation of specificity within the control group. Additional factors, such as stool storage conditions and DNA degradation rates, may influence these results, necessitating further investigation. Finally, a comprehensive discussion of the role of stool DNA testing in colorectal neoplasia screening requires evaluating factors beyond sensitivity and specificity, including test performance, intervals, complications, costs, and patient adherence, which are beyond the scope of this paper. In conclusion, the combined SDC2/SFRP2/TFPI2 methylation panel exhibited high sensitivity for colorectal neoplasia and outperformed conventional FOBT and serum CEA. When used in conjunction with FOBT and serum CEA, it yields the best results. This approach may serve as an important auxiliary or alternative to colonoscopy, offering a promising strategy for reducing the incidence and mortality of CRC through enhanced early detection and risk stratification.

## Data Availability

To protect patient privacy and confidentiality, all personal identifiers were removed from the datasets generated or analyzed during the current study. The de-identified relevant data are provided in the **Supplementary Files**.
