# Supplementary material for: Stool-based SDC2/SFRP2/TFPI2 methylation assay for colorectal neoplasia screening: a multicenter, case-control study
Source: Front Oncol. 2026 Jan 30;16:1748759. doi: 10.3389/fonc.2026.1748759 (PMC12904125; doi:10.3389/fonc.2026.1748759)
Supplement: Supplementary file 1 [file DataSheet1.docx]

Details of the Logistic Regression Prediction Model

In this study, a logistic regression model was developed to evaluate the diagnostic efficacy of detecting SDC2/SFRP2/TFPI2 methylation, conducting fecal occult blood tests (FOBT), and measuring serum carcinoembryonic antigen (CEA) levels, both individually and in combination, for colorectal cancer (CRC) diagnosis. The model's variables were defined as follows: the independent variables included the ΔCt value from the combined SDC2/SFRP2/TFPI2 methylation assay (a quantitative measure), FOBT results (a binary variable with positive coded as 1 and negative as 0), and serum CEA concentration (a binary variable). The dependent variable was the CRC diagnostic outcome, represented as a binary variable with the case group coded as 1 and the control group as 0, where the control group comprised healthy individuals and non-CRC subjects. Multivariate logistic regression analysis was employed for model construction, with variable selection executed through the stepwise regression method. The final regression coefficients were determined to be 5.221 for the combined SDC2/SFRP2/TFPI2 methylation assay, 2.514 for FOBT, and 1.693 for CEA, with a constant term coefficient of -3.887.

Following the completion of model construction, validation was conducted to ensure the model's stability and generalization capability. A random selection of 30% of the study samples constituted the validation set. The model's predictive efficacy was assessed using receiver operating characteristic (ROC) curve analysis, with the area under the curve (AUC) and 95% confidence interval (CI) calculated to quantify performance. Based on the model's regression coefficients, a risk scoring formula was developed: Risk Score = 5.221 × (quantitative value of combined SDC2/SFRP2/TFPI2 methylation assay) + 2.514 × (FOBT result) + 1.693 × (CEA concentration) - 3.887. This formula facilitates the calculation of an individual's probability of CRC and, when used in conjunction with the optimal cutoff value derived from the ROC curve, offers auxiliary support for clinical diagnostic decision-making.
